# Staphylococcal Superantigen-like protein 11 mediates neutrophil adhesion and motility arrest, a unique bacterial toxin action

**DOI:** 10.1038/s41598-019-40817-x

**Published:** 2019-03-12

**Authors:** Chen Chen, Chen Yang, Joseph T. Barbieri

**Affiliations:** Department of Microbiology and Immunology, Medical College of Wisconsin, Milwaukee, WI 53226 USA

## Abstract

Methicillin resistant *Staphylococcus aureus* (MRSA) is a major human pathogen, which causes superficial to lethal clinical infections. Neutrophils are the most abundant leukocytes in the blood and are the first defense mechanism against *S*. *aureus* infections. Here we show Staphylococcal Superantigen-Like protein 11 (SSL11) from MRSA USA300_FPR3757 mediated differentiated human neutrophil-like cells (dHL60) motility arrest by inducing cell adhesion and “locking” cells in adhesion stage, without inducing oxidative burst. Pre-incubation of SSL11 with the glycan Sialyl Lewis X blocked SSL11 function and de-glycosylation of dHL60 cells by PNGase F abolished SSL11 binding, suggesting that SSL11 functions via interacting with glycans. This is the first description of a bacterial toxin inhibiting neutrophil motility by inducing adhesion and “locking” cells in an adhesion stage. Therefore, this study might provide a new target against *S*. *aureus* infections.

## Introduction

*Staphylococcus aureus* (*S*. *aureus*) is a major opportunistic pathogen of humans, which causes superficial complications to lethal, invasive infections. Prevalence of methicillin-resistant S. aureus (MRSA) imposes a high burden on healthcare resources. USA300 is the predominant MRSA clone in the US^1^. *S*. *aureus* survival in humans requires evasion of the host immune system, where complement activation and neutrophil-mediated killing are the primary defense mechanisms^2^.

*S*. *aureus* Superantigen-Like proteins (SSLs) are not mitogenic to T cells and do not bind MHC class II molecule, despite sharing similar structure with Superantigens (SAgs)^3^. Not all SSLs functions are known, but SSL activities identified so far involve immune evasion: SSL3, SSL5 and SSL11 inhibit neutrophil activation and rolling^4–6^; SSL7 and SSL10 bind IgA and IgG and inhibits complement activation^7–10^. Soluble factor(s) from *Lactobacillus reuteri* (*L. reuteri*) RC-14 inhibits *S*. *aureus* infections in a rat surgical-implant model by inhibiting *S*. *aureus* adhesion to surgical implants^11^. SSL11 showed a dramatic decrease in expression when *S*. *aureus* was co-cultured with *L*. *reuteri* RC-14 and recombinant SSL11 reacted with all five convalescent human sera samples from patients with previous *S*. *aureus* infections^12^, suggesting that SSL11 plays an important role for *S*. *aureus* infections. Understanding immune modulating protein SSL11 from MRSA might provide new targets against *S*. *aureus* infections.

Neutrophils are the most abundant leukocytes and the first host immune defense against *S*. *aureus* infection. The evasion of host neutrophil recruitment to the site of infection is essential to the success of *S*. *aureus* as a pathogen^2^. Precise regulation of neutrophil adhesion and de-adhesion is essential for migration towards a site of inflammation^13^. Differentiated HL60 cells (dHL60) are a widely-used model of human neutrophils for migration and chemotaxis^14^. In the current study, we show for the first time that SSL11 disrupts neutrophil motility by induction of cell adhesion. These findings provide a new therapeutic target against *S*. *aureus* infections and neutrophil overstimulated inflammatory diseases.

## Results

### SSL11 induces dHL60 cells adhesion and “locks” cells in adhesion stage

**Figure 1:**
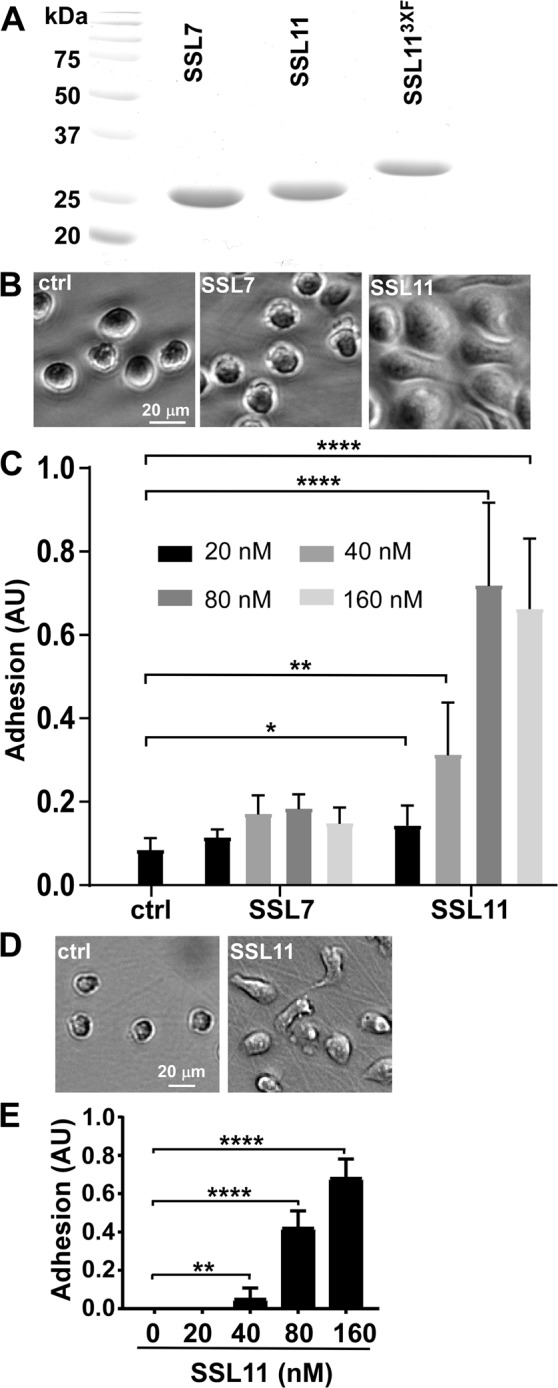
SSL11 stimulates dHL60 cell adhesion. (**A**) 2 μg of purified SSL7, SSL11 and SSL11^3XF^ were separated by SDS-PAGE and stained with Coomassie Blue. (**B**) dHL60 cells were incubated with 80 nM of SSL7 or SSL11 in fibronectin (FN)-coated plates at 37 °C for 30 min followed by two PBS washes. Representative DIC images were shown. (**C**) dHL60 cells were incubated with SSL7 or SSL11 in FN-coated 96-well plates at 37 °C for 30 min followed by two PBS washes. Adherent cells were quantified by crystal violet staining and shown as adhesion arbitrary unit (AU). (**D**) dHL60 cells were incubated with 80 nM of SSL11 at 37 °C for 30 min and chased in fresh media without SSL11 for another 4 hours in FN-coated plates. Representative DIC images were shown. (**E**) dHL60 cells were treated with SSL11 as described in (**D**) in FN-coated 96 well plates. Adherent cells were quantified by crystal violet staining and shown as adhesion arbitrary unit (AU).

In humans, *S*. *aureus* survives host immune system by evasion of complement activation and neutrophil-mediated killing^2,15^. Relative to primary neutrophils, differentiated human HL60 cells (dHL60) are more homogeneous, stable, and more efficient for genetic manipulation. As suspension cells, quiescent dHL60 cells display low adherence. After 30-min incubation with SSL11, dHL60 cells transitioned from a non-adhesion to an adhesion phenotype, while untreated cells remained non-adhesion (Fig. 1A,B). A quantitative plate assay showed that SSL11 induced dHL60 cell adhesion in a dose-dependent manner, with 40 nM SSL11 inducing about 50% cell adhesion (Fig. 1C). SSL11 induced adhesion as early as 5 min, with >75% cell adhesion detected by 15 min (Supplementary Fig. S1, Movie 1 and 2). SSL7, which binds IgA and IgG, inhibits complement activation^7,9,10^, did not mediate dHL60 cell adhesion (Fig. 1B,C), showing the specificity of SSL11-mediated cell adhesion. To test how long cells remained adhesive after SSL11 treatment, dHL60 cells were incubated with SSL11 for 30 mins, and cells were chased in media without SSL11 for another four hours. Unexpectedly, dHL60 cells remained adhesive four hours later in a dose-dependent manner, suggesting that SSL11 “locked” cells in adhesion stage (Fig. 1D,E). SSL11 is the first known member of the SSL family to induce cell adhesion.

### SSL11 inhibits fMLP-mediated dHL60 cells motility

**Figure 2:**
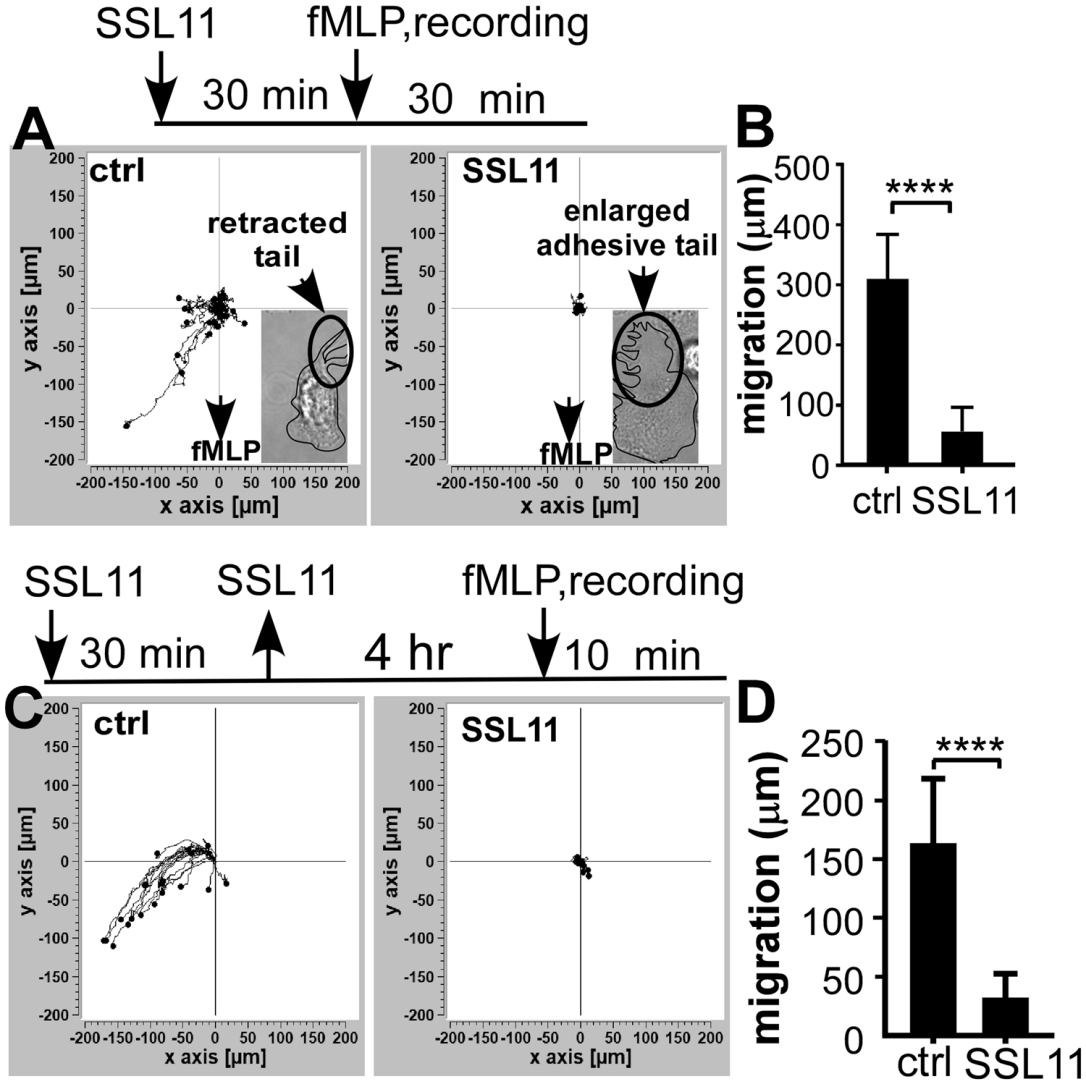
SSL11 blocks fMLP-induced dHL60 cell motility. (**A**) dHL60 cells were incubated with 80 nM of SSL11 at 37 °C for 30 mins in FN-coated plates when fMLP was added and cell motility was recorded for 30 min. Representative motility traces of 20 cells were shown as individual tracks using Ibidi Chemotaxis and Migration Tool. Inserts show hand traced outline of representative images of ctrl cell (small tail) and SSL11-stimulated cell (enlarged adhesive tail). (**B**) Total length of motility of the 20 cells (μm) shown in (**A**) was plotted by GraphPad Prism. (**C**) dHL60 cells were incubated with SSL11 at 37 °C for 30 mins in Fn-coated plates and chased in fresh media without SSL11 for another 4 hrs when fMLP was added and cell motility was recorded for 10 min. Representative motility traces of 20 cells were shown as individual tracks. (**D**) Total length of motility of the 20 cells (μm) shown in (**C**) was plotted by GraphPad Prism.

Neutrophil migration requires a well-regulated balance between adhesion and de-adhesion, where interruption of this balance affects neutrophil motility. To test if SSL11-mediated dHL60 cell adhesion affects cell motility, the effect of SSL11 on chemotactic peptide fMLP-induced cell motility was tested. fMLP was added to the edge of a fibronectin (FN)-coated well and cell motility was recorded for 30 mins. Upon the addition of fMLP, dHL60 cells migrated an average length of 307 μm, while cells pre-incubated with SSL11 migrated an average length of 55 μm (Fig. 2A,B). Visualization revealed that SSL11 pre-incubated dHL60 cells responded to fMLP stimulation, but did not migrate and appeared to have enlarged adhesive tails (Fig. 2A insert, Supplementary Movies 3 and 4). Thus, SSL11 did not inhibit cell motility by directly interfering with fMLP sensing, but rather, SSL11 inhibited cell motility by increasing tail-localized cell adhesion. Moreover, SSL11-treated cells displayed minimum migration toward fMLP even four hours after SSL11 was taken out, suggesting that “locking” cells in adhesion stage by SSL11 abolished cell motility (Fig. 2C,D).

### Dose-dependent SSL11 association with dHL60 cells

**Figure 3:**
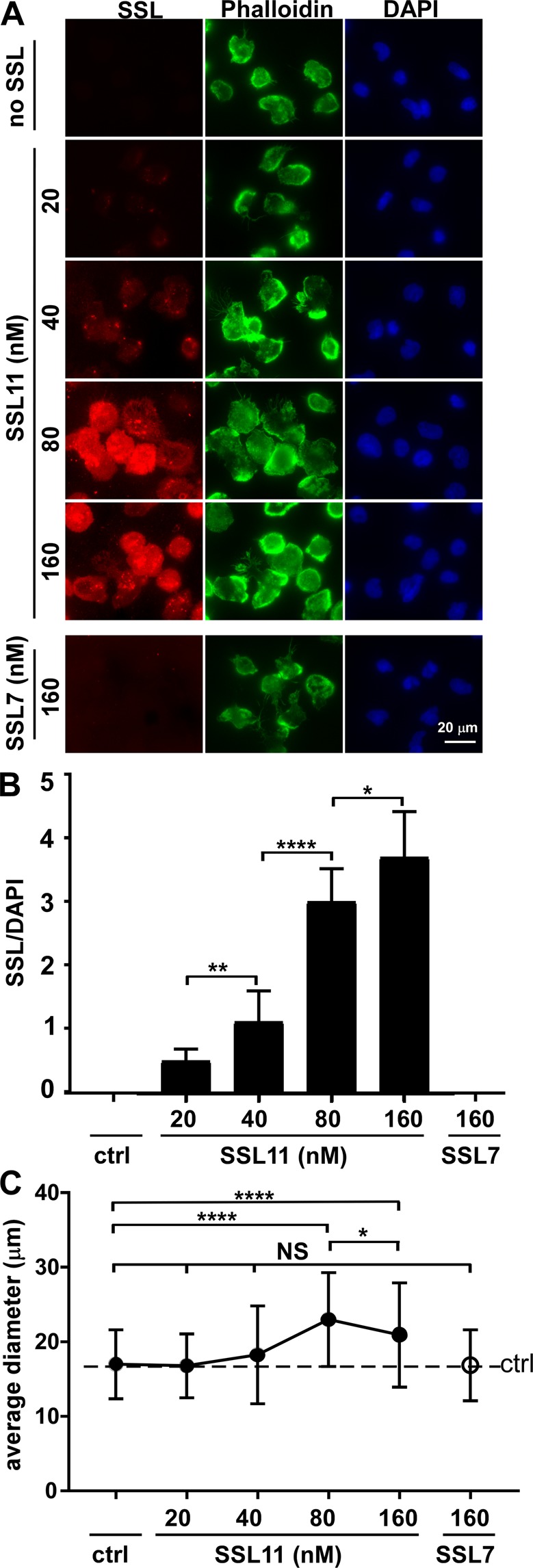
Dose-dependent SSL11 association with dHL60 cells. (**A**) dHL60 cells were incubated with SSL7^568^ or SSL11^568^ (20–160 nM) in FN-coated plates at 37 °C for 30 min. Cells were washed, fixed, and incubated with Phalloidin^647^ at room temperature for one hour followed by DAPI staining. Cell staining is shown: SSL (red), Phalloidin (green), and DAPI (blue). (**B**) Quantification of SSL association with dHL60 cells. Fluorescence intensity of SSL/DAPI was quantified by ImageJ. (**C**) The diameters (μm) of 100 random cells were measured by ImageJ and plotted by GraphPad Prism.

SSL11 association with dHL60 cells was dose-dependent and correlated with cell adhesion (Fig. 3A,B). Cell adhesion was detected at 40 nM SSL11 and reached maximum adhesion at 80 nM SSL11 (Fig. 1C). SSL7 did not bind to dHL60 cells (Fig. 3A,B) and did not induce cell adhesion (Fig. 1C), supporting that cell association is required for SSL11-induced cell adhesion and motility arrest. In addition, SSL11-induced cell adhesion was associated with cell spreading when the average cell diameter was quantified (Fig. 3C).

### SSL11 induces human monocytic cell line THP-1 cells spreading

**Figure 4:**
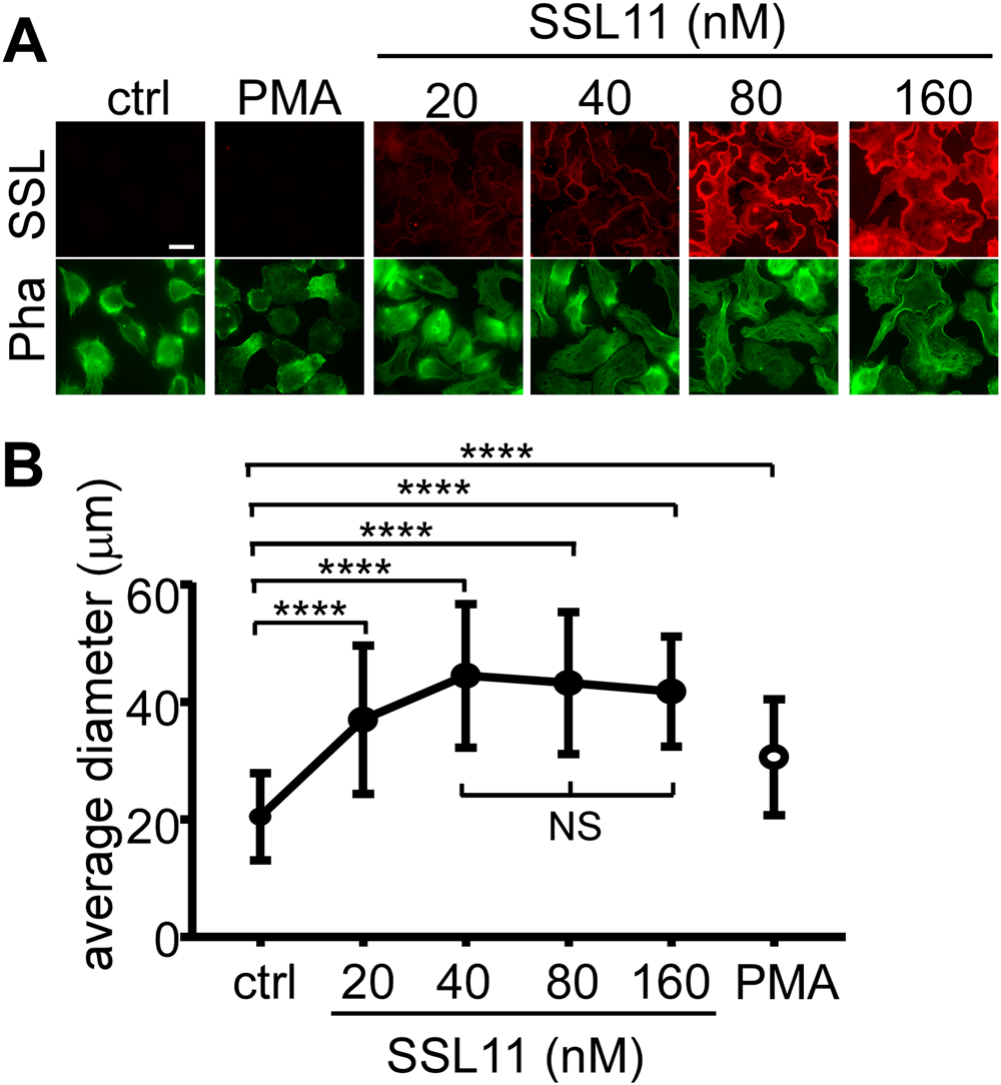
SSL11 induced human monocytic cell line THP-1 cells spreading. (**A**) THP-1 cells were incubated with either PMA (100 ng/ml) or indicated amount of SSL11^568^ at 37 °C for 30 min. Cells were washed, fixed, and incubated with Phalloidin^647^ at RT for one hour followed by DAPI staining. Cell staining is shown: SSL (red) and Phalloidin (green). Scale bar: 10 μm. (**B**) The diameters (μm) of 100 random cells were measured and plotted by GraphPad Prism.

To test if SSL11 functions on other cell types, THP-1, a human monocytic cell line, was incubated with either Phorbol 12-Myristate 13-Acetate (PMA, positive control) or SSL11^568^. Like dHL60 cells, SSL11 bound and induced THP-1 cell spreading in a dose-dependent manner when the average cell diameter was quantified (Fig. 4).

### Glycan-dependent SSL11 function

**Figure 5:**
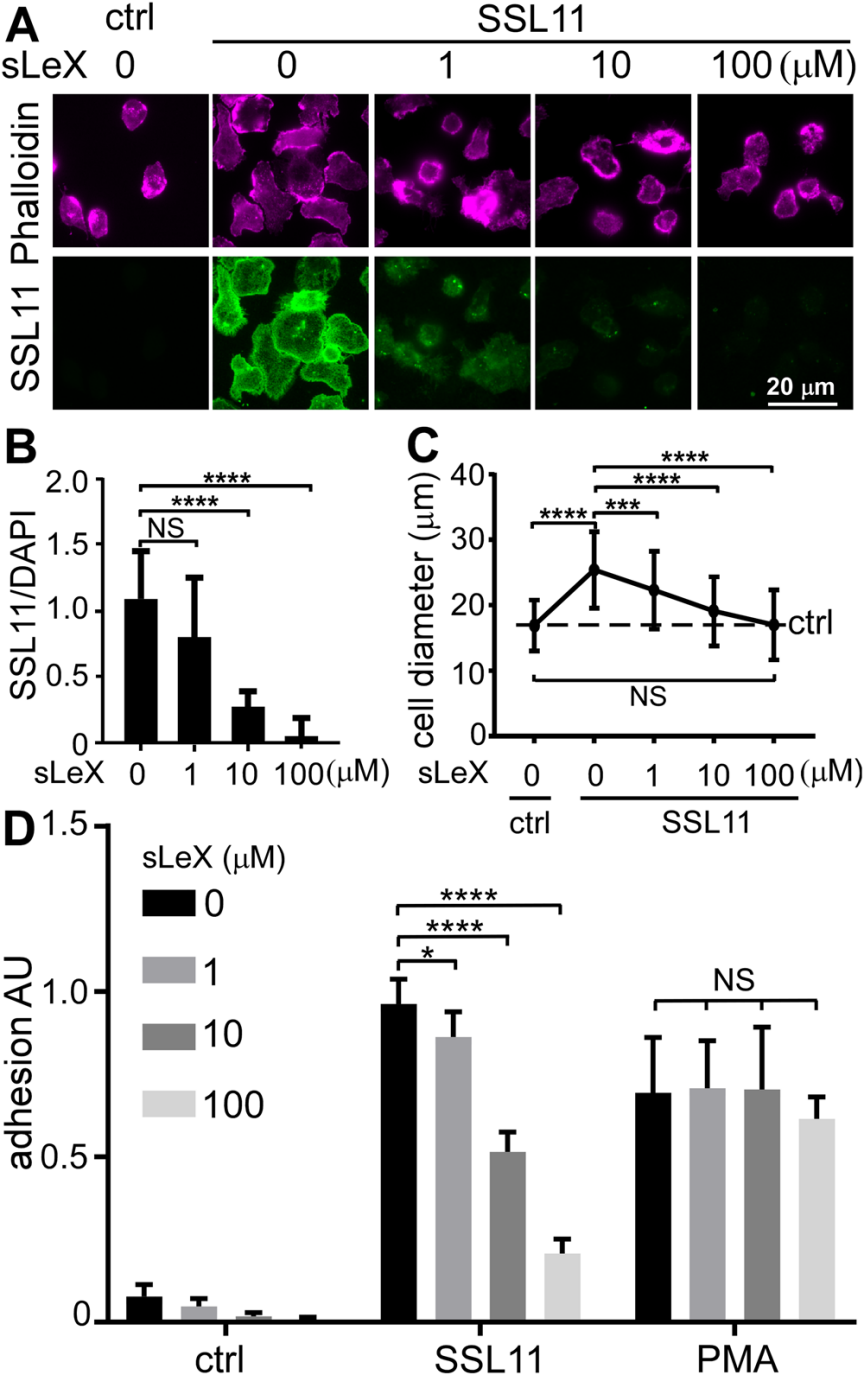
SLeX inhibits SSL11 cell association and SSL11-induced cell spreading and adhesion. (**A**) SSL11^568^was pre-incubated with SLeX at 4 °C for 2 hrs before incubated with dHL60 cells at 37 °C for 30 min. Cells were washed and fixed. Fixed cells were incubated with Phalloidin^647^ at RT for one hour followed by DAPI staining. Cell staining is shown: SSL (green), Phalloidin (magenta). (**B**) Quantification of SSL association with dHL60 cells. Fluorescence intensity of SSL/DAPI was quantified by ImageJ. (**C**) The diameters (μm) of 100 random cells were measured and plotted by GraphPad Prism. (**D**) SSL11 and PMA were pre-incubated with SLeX at 4 °C for 2 hrs before incubation with dHL60 cells in FN-coated 96-well plates at 37 °C for 30 min followed by two PBS washes. Adherent cells were quantified by crystal violet staining and shown as adhesion AU.

SSLs are divided into two subgroups depending on the presence of a Sialyl Lewis X [SLex–Neu5Aca2-3Galb1-4(Fuc1-3) GlcNAc, SLeX] binding site within the C-terminal domain. SSL2-6 and SSL11 possess this conserved carbohydrate binding motif^4,5^. To test whether SSL11-induced cell adhesion and motility arrest is glycan dependent, SSL11 was pre-incubated with exogenous SLeX before incubation with dHL60 cells. SLeX inhibited SSL11 cell association and SSL11-induced cell spreading, adhesion in a dose-dependent manner with complete inhibition at 100 μM. As a control, PMA-induced cell adhesion was not affected by SLeX, suggesting that SSL11 binds and functions via interacting with SLeX specifically (Fig. 5). SLeX is the sub-terminal disaccharide *N*-acetyllactosamine (LacNAc) on both *N*- and *O*-glycans. N-linked glycosylation is the most common form of covalent protein modification in human cells, especially for secretory and membrane-bound glycoproteins^16^. To further test whether SSL11 binding and function are glycan-dependent, dHL60 cells lysate were treated with PNGase F to remove N-linked glycosylation, which was confirmed by loss of Erythrina Cristagalli Lectin (ECL) binding and Silver staining (Supplementary Fig. S2A,B). A 3XFLAG epitope tag was engineered at the N terminus of SSL11 (SSL11^3XF^) (Fig. 1A) and SSL11^3XF^ induced dHL60 cell adhesion, similar to non-FLAG tagged SSL11 (Supplementary Fig. S3). SSL11^3XF^ bound to dHL60 cell lysate at a region of 75–150 kDa by Far Western blotting and de-glycosylation by PNGase F abolished those interactions (Supplementary Fig. S2C). This further suggests that SSL11 binding and stimulation of neutrophil adhesion are glycan-dependent.

### SSL11 does not induce oxidative burst in dHL60 cells

**Figure 6:**
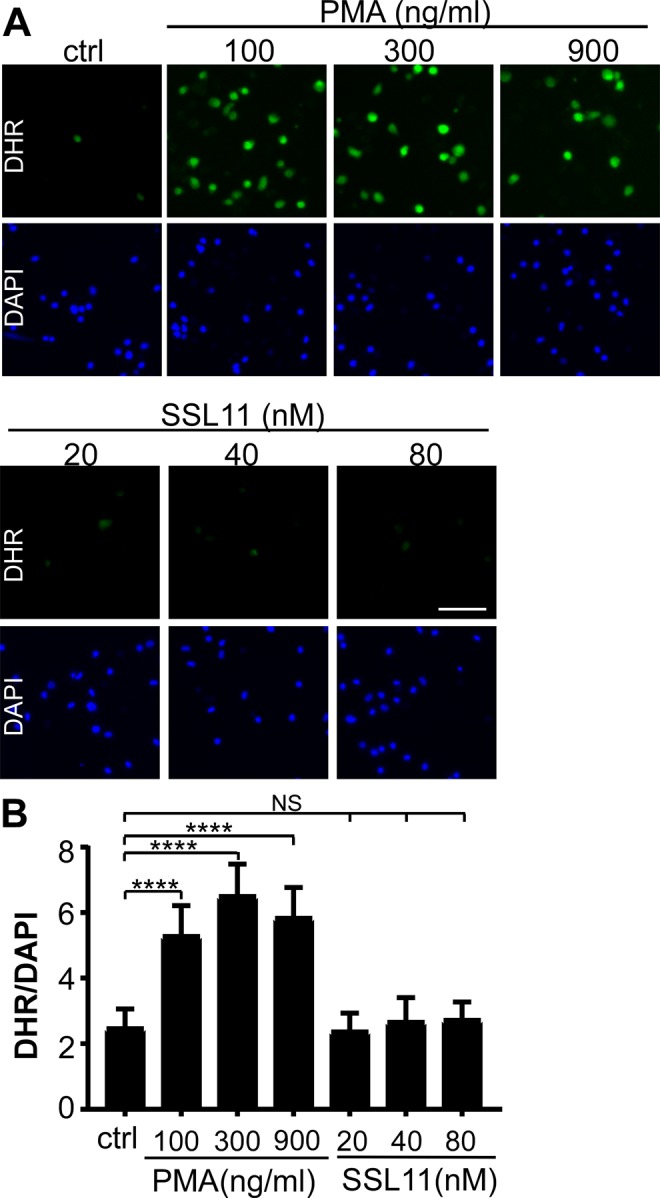
SSL11 did not induced dHL60 cells oxidative burst by DHR123 assay. (**A**) dHL60 cells were incubated with 25 μM DHR123 in serum free medium at 37 °C for 30 min followed by incubation with PMA or SSL11 at 37 °C for 30 min. Cells were washed, fixed and stained by DAPI. DHR123 fluorescence was observed under microscopy using FITC channel. Cell staining is shown: oxidized DHR123 by reactive oxygen species (ROS) (green) and DAPI (blue). Scale bar: 40 μm. (**B**) Quantification of SSL association with dHL60 cells. Fluorescence intensity of DHR123/DAPI was quantified by ImageJ.

Oxidative burst describes the phagocytic response of cells to produce reactive oxygen species (ROS). The uncharged and nonfluorescent Dihydrorhodamine 123 (DHR123) is a widely used ROS indicator which can passively diffuse across membranes. When oxidized, DHR123 is converted to rhodamine123 which fluoresces when excited at 488 nm. The oxidative burst of neutrophils can be triggered by PMA^17^. To test if SSL11 induced oxidative burst of dHL60 cells, dHL60 cells were pre-incubated with DHR123 followed by incubation with either PMA or SSL11. PMA induced strong ROS production shown by green fluorescence while SSL11 did not (Fig. 6), suggesting that SSL11 did not activate dHL60 cells oxidative burst although SSL11 induced cell adhesion and spreading.

### SSL11 does not induce cell adhesion via PSGL-1

**Figure 7:**
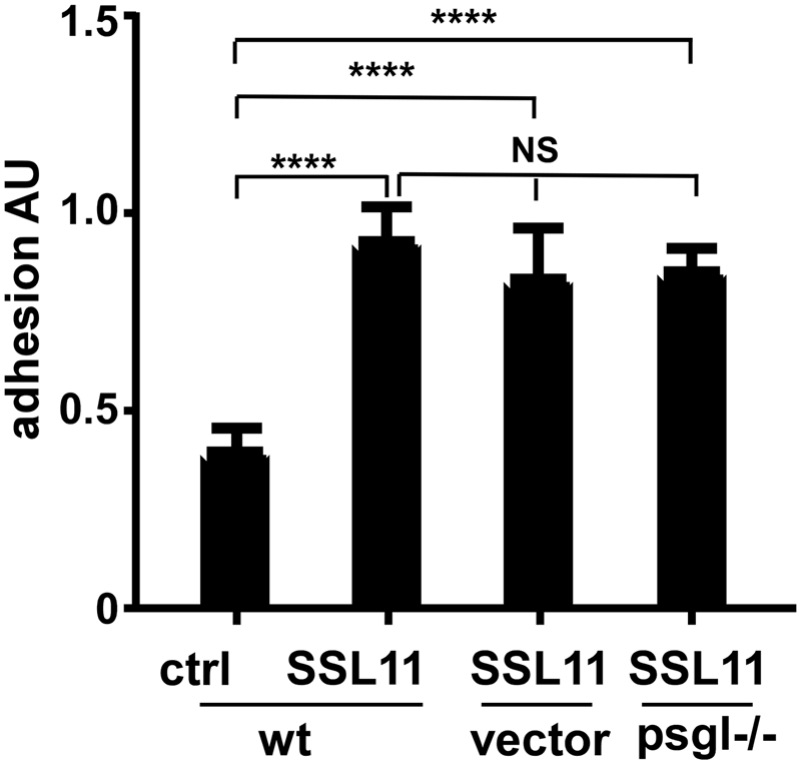
SSL11 induces cell adhesion independent of PSGL-1. *wt*, vector control and *psgl−/−* dHL 60 cells were incubated alone (ctrl) or with 80 nM of SSL11 in FN-coated 96-well plates at 37 °C for 30 mins followed by two PBS washes. Adherent cells were quantified by crystal violet staining and shown as adhesion AU.

Earlier studies reported that SSL11 blocked neutrophil rolling by interacting with PSGL-1^4^. To test if PSGL-1 is also responsible for SSL11-induced cell adhesion and spreading, we generated a *psgl-1* knockout cell line using CRIPR-Cas9 system (Supplementary Fig. S4). When *wt*, vector control or *psgl−/−* dHL60 cells were incubated with SSL11, SSL11 induced cell adhesion was not affected in *psgl−/−* cells (Fig. 7). This indicated that SSL11-induced cell adhesion was independent of PSGL-1.

## Discussion

In the current study, SSL11 from USA300_FPR3757 arrested fMLP-mediated neutrophil like dHL60 cells motility by inducing adhesion and “locking” cells in adhesion stage (Supplementary Fig. S5). This is a new function of SSL family proteins and sheds light on the understanding of the virulence and pathogenesis of *S*. *aureus*.

*S*. *aureus* is a potent pathogen as approximately 30% of the human population is continuously colonized and the vast majority of *S*. *aureus* diseases occur in immune-competent individuals. To achieve this level of pathogenicity, *S*. *aureus* deploys an arsenal of immune-evasive strategies to manipulate neutrophil function. Neutrophils, the first line of defense against invading pathogens, are the most abundant leukocytes with more than 50% of the bone marrow devoted to neutrophil production. Neutrophil recruitment into inflamed tissue is a well-regulated cascade of events: rolling, adhesion to endothelial cells, post adhesion strengthening, crawling, and finally transmigration^13,18,19^. To prevent neutrophil recruitment, *S*. *aureus* devotes various secreted factors targeting early steps of neutrophil recruitment: rolling and chemokine signaling. SSL5 and extracellular adherence protein (Eap) interfere with neutrophil rolling by binding to PSGL-1 and ICAM-1, respectively. SSL10 inhibits CXCL12-mediated responses by targeting CXCR4^20^ and SSL3 blocks recognition of staphylococcal lipoproteins and peptidoglycan by binding to TLR2^6^. Chemotaxis-inhibitor protein of *S*. *aureus* (CHIPS) binds and inhibits formyl peptide receptor 1 (FPR1) and C5a receptor (C5aR)^21–23^. In the current study, SSL11 from *S*. *aureus* USA300_FPR3757 induced cell adhesion and arrested fMLP-stimulated cell motility. A previous study showed that SSL11 blocks neutrophil rolling by interacting with PSGL-1^4^. Our study showed that SSL11-induced cell adhesion was independent of PSGL-1 (Fig. 7). Thus, SSL11 has multiple functions toward neutrophil manipulation as SSL5 displays several immune evasion functions^5,24–27^. Moreover, SSL11-induced cell adhesion was observed over different surfaces: FN-coated, ICAM-1 coated, plastic and glass surfaces (data not shown), suggesting that SSL11 might trigger multiple pathways to induce strong cell adhesion, which needs further investigation. SSL11 did not induce oxidative burst in dHL60 cells (Fig. 6) although SSL11 induced cell adhesion, suggesting that SSL11 functions as immune “suppressor” instead of immune “activator” such as Superantigens.

SSL11 from the MRSA strain USA300_FPR3757 was used in our study. SSL11 sequences from multiple strains (Newman, USA300, MW2, N315 and US6610) were aligned using Clustal Omega. SSL11s from Newman and USA300_FPR3757 were identical, while SSL11 from MW2, N315 and US6610 shared 59%, 72% and 69%, respectively, primary amino acid identity with SSL11 from USA300, with most sequence variation in the N-terminal region (Supplementary Fig. S6). This is consistent with previous studies showing that SSL11 exists in several alleles with 54% identity among strains: MW2, Mu50, Col and N315^4^. Since Fraser group used SSL11-US6610, we tested SSL11-US6610 effects on dHL60 cells. Like SSL11-USA300_FPR3757, SSL11-US6610 mediated dHL60 cell adhesion (Supplementary Fig. S7), suggesting induction of cell adhesion is a common function of SSL11 alleles, since SSL11-US6610 and SSL11-USA300 share only 69% sequence identity.

SSL11 provides extra mechanism of abrogating neutrophil recruitment by inducing cell adhesion and “locking” cells in adhesion stage, especially for neutrophils already rolling, ready for next step during migration. Elicitation of neutrophil adhesion by SSL11 blocks neutrophil migration to the site of infection, which is beneficial for *S*. *aureus* infection. Besides neutrophils, SSL11 also induced cell spreading for THP-1 cells, a human monocytic cell line. This suggests that SSL11 targets other leukocytes, which is beneficial for *S*. *aureus* infection. To our knowledge, this is the first description of a bacterial toxin inhibiting neutrophil motility by inducing adhesion and “locking” cells in the adhesion stage.

In the current study, SSL11-induced cell adhesion and spreading was glycan dependent, as pre-incubation of SSL11 with SLeX abolished SSL11 association and its function (Fig. 5) and de-glycosylation of cell lysate abolished SSL11 binding (Supplementary Fig. S2). Therefore, glycan SLeX serve as a functional receptor for SSL11. SLeX functions as a receptor for *H*. *Pylori* adhesion SabA, which allows the bacteria to cling to the surface of the gastric cell^28^. SLeX is an invariant ligand for P-, E- and L-selectin. The selectins bind weakly to SLeX-like glycans but with high affinity to glycoprotein with SLeX such as PSGL-1^29^. This is consistent with previous work that SSL11 binds PSGL-1 via glycans^4^. SSL11^3XF^ bound to dHL60 cell lysate at a region of 75–150 kDa in a glycan-dependent manner (Supplementary Fig. S2C), suggesting glycoproteins with SLeX are receptors for SSL11. Besides PSGL-1, other receptors need to be identified. Sharing a SLeX binding pocket with SSL11, SSL5 binds to PSGL-1, GPIbα, and GPCRs via glycan binding^5,25,26^. There might be an additional receptor binding site on SSL11 which determines the binding and function specificity. Bacterial toxins sometimes employ dual-receptor binding for specificity. Botulinum neurotoxin A (BoNT/A) binds to a ganglioside and a synaptic vesicle protein SV2A/B/C, BoNT/B binds to a ganglioside and a synaptic vesicle protein Synaptotagmins I/II while Tetanus neurotoxin binds to dual-gangliosides^30,31^.

SSL11 demonstrated a new mechanism of neutrophil manipulation by bacterial toxins: inducing intense adhesion to arrest cell motility, which can prevent neutrophils from migrating to the site of infection. SSL11 might provide a new target against *S*. *aureus* infections.

## Materials and Methods

### Antibodies and reagents

Mouse anti-FLAG M2 antibody (Sigma), HRP-conjugated anti-FLAG antibody (Sigma), APC PSGL-1 antibody (FLEG) (ThermoFisher), Human Fibronectin (ThermoFisher), DMSO (Sigma), Phalloidin Alex647 (ThermoFisher), RIPA buffer (Sigma), Phorbol 12-Myristate 13-Acetate (PMA, sigma), SLeX (Sigma), Biotinylated ECL (Vector Laboratories), PNGase F (New England Biolabs), Dihydrorhodamine 123 (DHR123) (ThermoFisher), ProLong Gold Antifade Mountant (ThermoFisher), HRP-conjugated streptavidin (ThermoFisher).

### Plasmid construction

*E*. *coli* codon-optimized sequences of *S*. *aureus* strain USA300 _FPR3757 SSL7 (GenBank: ABD22785.1) and SSL11 (GenBank: ABD21156.1) were synthesized (IDT) and subcloned into a pET28a vector for expression. An N-terminal 3 × FLAG tag was engineered downstream of His_6_ tag for construct SSL11^3XF^. All constructs contain an N-terminal His_6_ tag for protein purification. Constructs were confirmed by DNA sequencing.

Protein expression and purification. Plasmids encoding SSL7, SSL11 and SSL11^3XF^ were transformed into *E*. *coli* BL21(DE3). Transformants were grown overnight on LB agar plates containing 50 µg of kanamycin/ml, which were the inoculums for liquid cultures (LB, 400 ml) containing the same antibiotic. Cells were cultured at 37 °C to an optical density at 600 nm (OD_600_) of 0.6 when T7 promoter expression was induced with 1 mM IPTG. Cells were cultured overnight at 250 rpm at 16 °C. Cells were pelleted and lysed with a French press and clarified by centrifugation. His_6_-tagged proteins were purified using Ni^2+^-nitrilotriacetic acid (NTA) resin (Qiagen). Purified SSL7 and SSL11 were dialyzed into 20 mM Tris buffer (pH 7.9) with 200 mM NaCl and 40% glycerol. Aliquots were stored at −20 °C.

### Protein labeling by Alexa Fluor

SSL7 and SSL11 were labeled using Alex Fluo568 Protein labeling kit (Invitrogen, A10238). SSL7 and SSL11 were dialyzed in PBS at 4 °C overnight. 50 μl of 1 M bicarbonate was added to 0.5 ml of 2 mg/ml SSL7 or SSL11 followed by transfer the protein solution to a vial of reactive dye. The reaction mixture was stirred for two hours at room temperature followed by dialysis in PBS at 4 °C overnight to eliminate free dyes. Labeled proteins were stored at 4 °C.

### Cell Culture

The HL60 cell (ATCC® CCL-240™) is from a patient with acute promyelocytic leukemia, which can be differentiated into neutrophil-like cells^14^. HL60 cells were cultured and maintained in RMPI medium with 10% FBS and 20 mM HEPES. HL60 cells were differentiated in complete medium with 1.3% DMSO as described by Fleck RA *et al*.^14^. Differentiated HL60 (dHL60) cells show ≥90% viability (trypan blue), ≥55% CD35 expression and ≤20% CD71 expression. Differentiation was confirmed by Flow cytometry using anti-CD11b, anti-CD35 and anti-CD71 antibodies (data not shown). dHL60 cells were used for experiments at day 6–8 after differentiation. The human monocytic cell line THP-1 is a generous gift from Dr. Thomas Zahrt at Medical College of Wisconsin. THP-1 cells were cultured and maintained in RMPI medium with 10% FBS and 20 mM HEPES.

### dHL60 cells adhesion assay

96-well plates were coated with 100 μl of Fibronectin (10 μg/ml) in PBS at 4 °C overnight followed by two PBS washes. Adhesion assay was performed as previous described with modification^32^. dHL60 cells (3 × 10^5^ cells/well) were incubated with SSL7 or SSL11 (20–160 nM) at 37 °C for 30 min followed by two PBS washes. Adherent cells were incubated with 0.5% crystal violet at RT for 10 min followed by four PBS washes. The plate was dried and followed by addition of ethanol to solubilize cell bound crystal violet, absorbance at 595 nm was measured to quantify crystal violet and shown as adhesion arbitrary unit (AU). For SLeX blocking experiment, indicated amount of SLeX was incubated with SSL11 or PMA (10 ng/ml) in serum free RPMI media at 4 °C for two hours before incubation with dHL60 cells.

### dHL60 cells motility assay

dHL60 cells (5 × 10^5^ cells) were incubated with 80 nM of SSL11 at 37 °C for 30 min. 10 μM fMLP was added and cell motility was recorded by taking DIC images every 10 s for 30 min on a 37 °C heated stage. Twenty cells from each group were tracked using ImageJ “Manual tracking” and data were imported to Ibidi Chemotaxis and Migration Tool and plotted by GraphPad Prism 7.03 to show the individual cell moving tracks and total migration length (μm).

### SSL11 association with dHL60 cells

24-well plates with glass cover slips were coated with Fibronectin (10 μg/ml) in PBS at 4 °C overnight followed by two PBS washes. dHL60 cells (5 × 10^5^ cells/well) or THP-1 cells (2 × 10^5^ cells/well) were incubated with SSL7^568^ or SSL11^568^ (20–160 nM) at 37 °C for 30 min. Cells were washed twice with PBS and fixed with 4% paraformaldehyde at room temperature for 15 min. Cells were incubated in blocking solution (DPBS with 10% FBS, 2.5% cold-water fish skin gelatin, 0.1% Triton-X, and 0.05% Tween 20) for one hour followed by incubation with Phalloidin^647^ in incubation solution (DPBS 5% FBS, 1% cold-water fish skin gelatin, 0.1% Triton X, and 0.05% Tween 20) at RT for one hour. Cells were washed and incubated with DAPI for nuclei staining, then were fixed again with 4% paraformaldehyde at room temperature for 15 min. After wash, cover slips were mounted using ProLong Gold Antifade Mountant). For SLeX blocking experiment, indicated amount of SLeX was incubated with SSL11 or PMA (10 ng/ml) in serum free RPMI media at 4 °C for two hours before incubation with dHL60 cells. Images were captured with a Nikon TE2000 microscope using a Photometrics CoolSnap HQ2 camera. Images were captured by epifluorescence with a Sedat Quad cube (Chroma Technology Corp).

### Oxidative burst by DHR123 assay

dHL60 cells (5 × 10^5^ cells/well) were incubated with 25 μM DHR123 in serum free medium in glass bottom 24-well plates at 37 °C for 30 mins followed by incubation with PMA (100, 300 and 900 ng/ml) or SSL11 (20–80 nM) at 37 °C for 30 mins. Cells were washed, fixed and stained by DAPI. Dihydrorhodamine 123 is a nonfluorescent reactive oxygen species (ROS) indicator and is oxidized by ROS to exhibit green fluorescence. DHR123 fluorescence was observed under microscopy using FITC channel. Cell staining is shown: oxidized DHR123 by ROS (green) and DAPI (blue).

### Psgl-1 gene knockout by CRIPR-Cas9 system

Psgl-1 (SELPLG, NM_003006.4) gRNA CCTGCTGCAAGGCGTTCTAC was synthesized in the vector pSpCas9(BB)-2A-GFP (PX458) by GenScript (NJ, USA). Plasmids were transformed into *stbl3* competent cells (ThermoFisher) and purified using PureLink™ HiPure Plasmid Filter Maxiprep Kit (ThermoFisher). Vector control or psgl-1 plasmid (10μg) was delivered to dHL60 cells (10 × 10^6^) by electroporation using Lonza Nucleofector 2b using program Y-019. Cells were incubated at 37 °C overnight followed by flow cytometry single-cell sorting of GFP positive cells into 96-well plates. GFP positive cells were cultured in RPMI media with 20% FBS until clones were established. To validate the expression of PSGL-1, cells were incubated with APC PSGL-1 antibody at 4 °C for one hr in PBS with 10% FBS, followed by two washes in PBS with 2% FBS. Fluorescence was measured by BD FACSCalibur flow cytometry.

### Far Western Blotting

dHL60 cells were lysed using RIPA buffer and the dHL60 cell lysate was quantified using BCA assay. For de-glycosylation, dHL60 cell lysate were incubated with PNGase F at 37 °C for 24 hours. dHL60 cell lysate (2 μg) with or without PNGase F treatment were separated on SDS-PAGE and were transferred to PVDF membrane in duplicate. One membrane was blocked with 5% BSA, followed by incubation with biotinytlated ECL at RT for two hours and incubation with HRP-conjugated streptavidin for another one hour. The other membrane was blocked with 2% milk and followed by incubation with 10 nM SSL11^3XF^ and HRP-conjugated anti-FLAG M2 antibody at room temperature for 2 h. Both membranes were washed, incubated with SuperSignal, and images were taken with a Fluorchem HD2 camera system.

### Silver Staining

Gels from SDS-PAGE were fixed with 30% isopropanol, 10% glacial acetic acid, washed with water for 10 min for three times. Gel was incubated with 0.02% sodium thiosulfate for 90 s followed by two quick water rinses. The gel was incubated with 0.2% silver nitrate for 20 min followed by two quick water rinses. The gel was developed in develop buffer (6 g sodium carbonate, 50μl formaldehyde, and 3 ml of 0.2% sodium thiosulfate in 100 ml) as needed and was quenched using 10% acetic acid.

### Data analysis and statistics

Images were generated with equal exposure times and conditions. Image intensity analysis was performed using ImageJ (NIH). Figures were compiled using Canvas X 2017 (ACD Systems). Data were shown as means with standard errors of the means (SEM). Data were analyzed by unpaired two-tailed Student’s *t* test using GraphPad Prism 7.03 from three independent experiments. *P* values of <0.05 at the 95% confidence level are indicated by *, *P* values of <0.005 are indicated by **P values of <0.001 are indicated by *** and *P* values of <0.0001 are indicated by ****.

## Supplementary information

Staphylococcal Superantigen-like protein 11 mediates neutrophil adhesion and motility arrest, a unique bacterial toxin action

Supplementary Movie 1.

Supplementary Movie 2

Supplementary Movie 3

Supplementary Movie 4
