# Supplementary material for: Staphylococcal Superantigen-like protein 11 mediates neutrophil adhesion and motility arrest, a unique bacterial toxin action
Source: Sci Rep. 2019 Mar 12;9:4211. doi: 10.1038/s41598-019-40817-x (PMC6414612; doi:10.1038/s41598-019-40817-x)
Supplement: Supplementary file 1 — Staphylococcal Superantigen-like protein 11 mediates neutrophil adhesion and motility arrest, a unique bacterial toxin action [file 41598_2019_40817_MOESM1_ESM.pdf]

Staphylococcal Superantigen-like protein 11 mediates neutrophil adhesion and motility arrest, a unique bacterial toxin action

Chen Chen*, Chen Yang, Joseph T. Barbieri

Supplementary materials

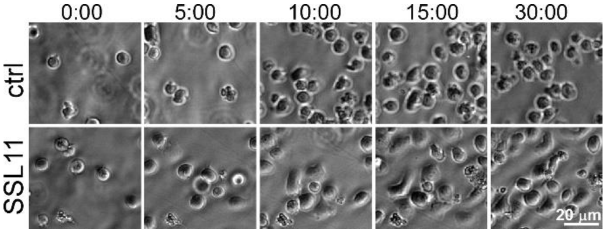

Fig. S1. **Time course of SSL11 induced dHL-60 cells adhesion.** dHL60 cells were incubated alone or with 80 nM of SSL11 for the indicated time (min).

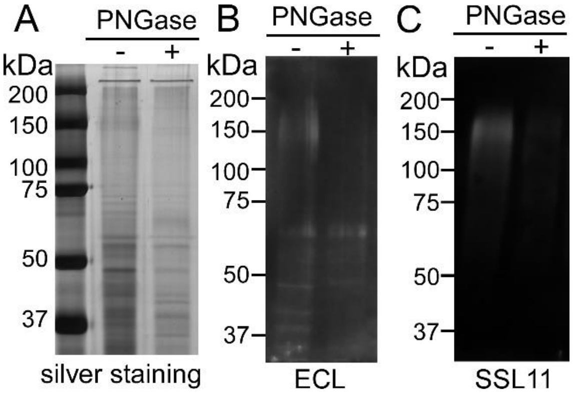

Figure S2. SSL11 binds to glycoproteins of HL60 cell lysate.
A. dHL60 cell lysate (2 µg) was treated with or without PNGase F followed by SDS-PAGE in triplicate. One gel was visualized by Silver staining. **B**. Two gels were transferred to PVDF membranes. One PVDF membrane was incubated with Biotinylated ECL followed by HRP-conjugated Streptavidin antibody and then incubated with Super Signal. **C**. The other PVDF membrane was subjected to Far Western blotting, using 10 nM of $SSL11^{3XF}$ bound to HRP-conjugated M2 anti-FLAG antibody and then incubated with Super Signal.

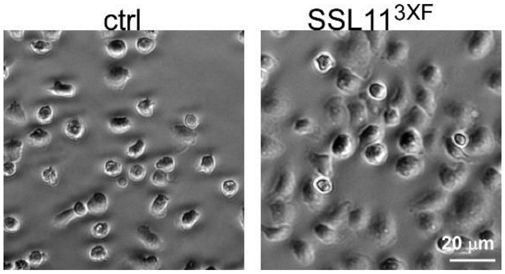

Fig. S3. SSL11^{3XF} stimulates dHL60 cell adhesion. dHL60 cells were incubated alone (ctrl) or with 80nM of SSL11^{3XF} at 37 °C for 30 min. Representative DIC images were taken after a PBS wash.

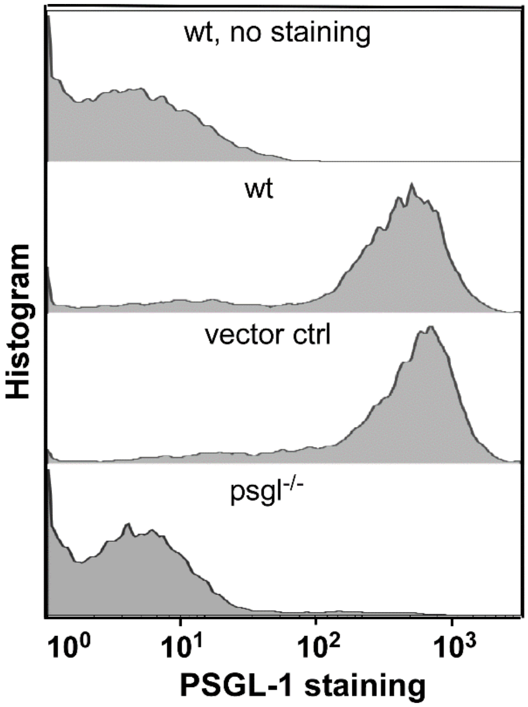

Fig. S4. Knockout psgl-1 gene by CRISPR-Cas9 in dHL60 cells. wt, vector control and psgl-/- dHL60 cells were incubated with APC labeled PSGL-1 antibody at 4 °C for one hr, washed, and the fluorescence was measured by flow cytometry. Histograms show PSGL-1 staining for wild type dHL60 (wt), dHL60 cells containing a vector control (vector ctrl) or dHL60 cells psgl-/- (psgl-/-). wt dHL60 cells without staining served as a control (wt, no staining).

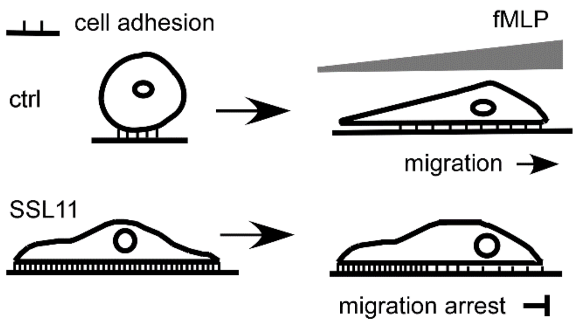

Fig. S5. Model illustrating SSL11-mediated neutrophil adhesion and motility arrest. Without stimulation, neutrophils show minimum adhesion on Fn surface. With fMLP stimulation, neutrophils polarize and migrate by balancing between adhesion and de-adhesion. Neutrophils spread upon incubation with SSL11. With fMLP stimulation, SSL11-treated neutrophils show a leading front, but migration is arrested due to an adhesive tail.

```
Newman  STLEVRSQATQDLSEYYNRPFFEYTNQSGY
USA300  STLEVRSQATQDLSEYYNRPFFEYTNQSGY
MW2     SRLSVTSKDTQELKKYYSGTGYNFQNVSGY
N315    STLEVRSQATQDLSEYYKGRGFELTNVTGY
US6610  STLEVRSQATQDLSEYYNRPYFDLRNLSGY
        * *.* *: **:*.:**.   ::  * :**

Newman  KEEGKVTFTPNYQLIDVTLTGNEKQNFG--
USA300  KEEGKVTFTPNYQLIDVTLTGNEKQNFG--
MW2     REGNKMNIIDGPQLNVVTLLGTDKERFKDD
N315    KYGNKVTFIDNSQQIDVTLTGNEKLTVKDD
US6610  REGNTVTFINHYQQTDVKLEGKDKDKIKDG
        :  ..:.:    *   *.* *.:*  .

Newman  EDISNVDIFVVRENSDRSGNTASIGGITKT
USA300  EDISNVDIFVVRENSDRSGNTASIGGITKT
MW2     EDYEGLDVFVVREGSGKHADNISIGGITKT
N315    DEVSNVDVFVVREGSDKSAITTSIGGITKT
US6610  -NNENLDVFVVREGSGRQADNNSIGGITKT
         : ..:*:*****.*.: . . ********

Newman  NGSNYIDKVKDVNLIITKNIDSVTSTSTSS
USA300  NGSNYIDKVKDVNLIITKNIDSVTSTSTSS
MW2     NKNQYKDPVQNVNLLTSKSNGQNTASVTSE
N315    NGTQHKDTVQNVNLSVSKSTGQHTTSVTSE
US6610  NRTQHIDTVQNVNLLVSKSTGQHTTSVTST
        * .:: * *::***  :*. .. *:: **

Newman  TYTINKEEISLKELDFKLRKHLIDKHNLYK
USA300  TYTINKEEISLKELDFKLRKHLIDKHNLYK
MW2     YYSINKEEISLKELDFKLRKQLIDKHDLYK
N315    YYSIYKEEISLKELDFKLRKHLIDKHDLYK
US6610  NYSIYKEEISLKELDFKLRKHLIDKHDLYK
         *:* ***************:*****:***

Newman  TEPKDSKIRITMKDGGFYTFELNKKLQTHR
USA300  TEPKDSKIRITMKDGGFYTFELNKKLQTHR
MW2     TEPKDSKIKVSMKNGGYYTFELNKKLQPHR
N315    TEPKDSKIRITMKNGGYYTFELNKKLQPHR
US6610  TEPKDSKIRVTMKNGDFYTFELNKKLQTHR
        ********:::**:*.:********** **

Newman  MGDVIDGRNIEKIEVNL
USA300  MGDVIDGRNIEKIEVNL
MW2     MGDTIDSRNIKKIEVNL
N315    MGDTIDSRNIEKIEVNL
US6610  MGDVIDGRNIEKIEVNL
        ***.**.***:******
```

SSL11 Strains	Newman	USA300	MW2	N315	US6610
% Identity to USA300	100	–	59	72	69

Fig. S6. SSL11 sequence alignments from five *S. aureus* strains. Primary amino acid sequence of SSL11 from *S. aureus* MW2, Newman, USA300_FPR3757, N315 and US6610 were aligned by Clustal Omega. A conserved Sialyl Lewis X binding pocket among the SSL11 isolates is highlighted in grey background. SSL11 Sequence identities compared to strain USA300_FPR3757: MW2 59%, USA300 100%, N315 72% and US6610 69%.

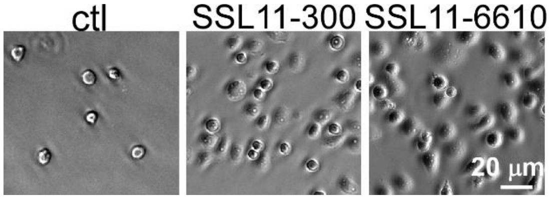

Fig. S7. SSL11-USA300_FPR3757 and SSL11-US6610 mediate cell adhesion. dHL60 cells were incubated with 80 nM SSL11-USA300_FPR3757 or SSL11-US6610 in fibronectin coated plate at 37 °C for 30 min. Cells were fixed and representative images (light microscopy) are shown.

Supplementary Movie 1. Control cells 30 mins. dHL60 cells were incubated alone in Fn-coated plate and was recorded by taking DIC images every 10 sec for 30 mins on a 37 °C heated stage.

Supplementary Movie 2. SSL11 induced dHL-60 cells adhesion. dHL60 cells were incubated with 80 nM of SSL11 in Fn-coated plate and was recorded by taking DIC images every 10 sec for 30 mins on a 37 °C heated stage.

Supplementary Movie 3. fMLP-induced dHL60 cell motility. 10 μM of fMLP was added to dHL60 cells in Fn-coated plate and cell motility was recorded by taking DIC images every 10 sec for 30 mins on a 37 °C heated stage.

Supplementary Movie 4. SSL11 blocks fMLP-induced dHL60 cell motility. dHL60 cells were incubated with 80 nM of SSL11 in Fn-coated plate at 37°C for 30 mins. 10 μM of fMLP was added and cell motility was recorded by taking DIC images every 10 sec for 30 mins on a 37 °C heated stage.
